# 

**Published:** 1868-03

**Authors:** 


The Annual meeting of the Northern Ohio Dental Association will be held in the Lecture Room of the Charity Hospital Medical College, at Cleveland, on Tuesday, May 5th, 1868, at 10 o'clock, A. M.

Subjects for discussion: 1. Reading of Essays. 2. Manipulation of gold foil in filling Teeth. 3. Fillings of different metals approximating each other. 4. Use of the mallet in filling. 5. Improvements in Dental Mechanism.

B. F. ROBINSON, *President.*

C. R. Butler, *Cor. Sec.*

### CONTENTS.

### The Dental Register

### OF THE WEST.

*Issued Monthly, at $3 a Year in Advance.*

DEVOTED TO THE INTERESTS OF THE DENTAL PROFESSION.

EDITED BY J. TAFT AND G. WATT.

The volume commences in January and closes in December.

The Register being issued monthly is always fresh, therefore indispensable to the practitioner who desires to keep posted up with the new inventions and improvements which are daily developed. Neither expense nor effort will be spared to give value and variety to its contents. Articles will be illustrated with well executed engravings whenever they will add to the interest or assist in explanation.

Its extensive circulation and frequency of issue makes it one of the BEST DENTAL ADVERTISING MEDIUMS in the United States.

RATES OF ADVERTISING.

One page, 1 year, $50. Half page, 1 year, $30. Quarter page, or card, 1 year $20.

All advertising bills payable in advance, or at furthest after the issue of the first number containing the advertisement. All transient advertisements to be paid for in advance

☞ CONTRIBUTIONS SOLICITED.

Address, J. TAFT, or "DENTAL REGISTER," Cincinnati Ohio.

Business Communications to

J. TAFT, Publisher,

No. 238 Fourth St., between Plum and Central Avenue

